# Genetic Interactions Due to Constitutive and Inducible Gene Regulation Mediated by the Unfolded Protein Response in *C. elegans*

**DOI:** 10.1371/journal.pgen.0010037

**Published:** 2005-09-23

**Authors:** Xiaohua Shen, Ronald E Ellis, Kenjiro Sakaki, Randal J Kaufman

**Affiliations:** 1 Howard Hughes Medical Institute, Department of Biological Chemistry, University of Michigan Medical Center, Ann Arbor, Michigan, United States of America; 2 Department of Molecular Biology, The UMDNJ School of Osteopathic Medicine, Stratford, New Jersey, United States of America; Stanford University School of Medicine, United States of America

## Abstract

The unfolded protein response (UPR) is an adaptive signaling pathway utilized to sense and alleviate the stress of protein folding in the endoplasmic reticulum (ER). In mammals, the UPR is mediated through three proximal sensors PERK/PEK, IRE1, and ATF6. PERK/PEK is a protein kinase that phosphorylates the alpha subunit of eukaryotic translation initiation factor 2 to inhibit protein synthesis. Activation of IRE1 induces splicing of *XBP1* mRNA to produce a potent transcription factor. ATF6 is a transmembrane transcription factor that is activated by cleavage upon ER stress. We show that in *Caenorhabditis elegans,* deletion of either *ire-1* or *xbp-1* is synthetically lethal with deletion of either *atf-6* or *pek-1,* both producing a developmental arrest at larval stage 2. Therefore, in *C. elegans, atf-6* acts synergistically with *pek-1* to complement the developmental requirement for *ire-1* and *xbp-1*. Microarray analysis identified inducible UPR (i-UPR) genes, as well as numerous constitutive UPR (c-UPR) genes that require the ER stress transducers during normal development. Although *ire-1* and *xbp-1* together regulate transcription of most i-UPR genes, they are each required for expression of nonoverlapping sets of c-UPR genes, suggesting that they have distinct functions. Intriguingly, *C. elegans atf-6* regulates few i-UPR genes following ER stress, but is required for the expression of many c-UPR genes, indicating its importance during development and homeostasis. In contrast, *pek-1* is required for induction of approximately 23% of i-UPR genes but is dispensable for the c-UPR. As *pek-1* and *atf-6* mainly act through sets of nonoverlapping targets that are different from *ire-1* and *xbp-1* targets, at least two coordinated responses are required to alleviate ER stress by distinct mechanisms. Finally, our array study identified the liver-specific transcription factor CREBh as a novel UPR gene conserved during metazoan evolution.

## Synopsis

The endoplasmic reticulum (ER) is an intracellular organelle where proteins fold and assemble prior to transport to the cell surface. The ER contains a finely tuned quality control apparatus to ensure that improperly folded proteins are retained in the ER lumen. A variety of physiological demands, environmental perturbations, and pathological conditions compromise protein folding in the ER and lead to the accumulation of unfolded proteins. The unfolded protein response (UPR) is an evolutionarily conserved intracellular adaptive signaling pathway that alleviates protein-folding defects in the ER. The unfolded protein signal is transmitted from the ER to the nucleus by three pathways involving the proteins ATF-6, PEK-1, and IRE-1/XBP-1. However, it is not known how these three pathways coordinate downstream transcriptional activation to mediate either cell adaptation or cell death. The authors have studied the nematode *Caenorhabditis elegans* to present a comprehensive genetic and gene expression analysis of the three UPR pathways. The findings demonstrate that the UPR regulates the expression of hundreds of genes in the presence, as well as the absence, of ER stress in a manner that is more complex and diverse than previously known.

## Introduction

The endoplasmic reticulum (ER) is the primary site where all secretory and membrane proteins fold prior to transiting the secretory pathway. In addition, the ER is the major Ca^++^ storage organelle and the site of lipid and oligosaccharide synthesis [1]. Therefore, ER homeostasis is essential for cellular function and survival in all eukaryotes. The unfolded protein response (UPR) is a transcriptional and translational regulatory pathway that evolved to sense and alleviate protein-folding stress in the ER caused by physiological demands or environmental variation [2,3].

In yeast, the UPR is solely dependent on Ire1p [4,5]. Ire1p1 is a bifunctional protein kinase and endoribonuclease that cleaves an unconventional 252-base intron from *HAC1* mRNA, which encodes a basic leucine zipper (bZIP)–containing transcription factor. Elegant studies in *Saccharomyces cerevisiae* identified 381 UPR-inducible genes that function primarily in ER protein folding and trafficking, ER-associated degradation (ERAD), and phospholipid metabolism [6]. In mammals, two homologs of IRE1, IRE1α and IRE1β, exist, and both are able to cleave a 26-base intron in *XBP1* mRNA to create a translational frame-shift that alters the carboxyl terminus of the protein to produce a potent bZIP transcription factor [7–11]. The primary targets that require the IRE1/XBP1 pathway are genes encoding functions in ERAD, such as EDEM (ER degradation-enhancing α-mannosidase-like protein), which recognizes specific glycoforms on unfolded proteins and directs them to the 26S proteasome [12].

In mammals, two additional ER transmembrane proteins, PERK/PEK and ATF6 mediate the UPR [13,14]. PERK phosphorylates the α subunit of eukaryotic translation initiation factor 2 (eIF2α) to mediate translational attenuation [15,16]. In addition, eIF2α phosphorylation paradoxically increases translation of *ATF4* mRNA, which encodes a transcriptional activator required for induction of an anti-oxidative response and amino acid biosynthesis and transport functions [16,17]. However, induction of the protein chaperone BiP, a primary marker of UPR activation, remained intact in *ire1* ^−/−^ mouse embryonic fibroblasts (MEFs) [18] and was only partially reduced in *perk^−/−^* MEFs and in eIF2α-phosphorylation-resistant MEFs [16,19], suggesting the existence of another UPR signaling pathway in mammals. ATF6 p90 is a type II transmembrane protein that contains a bZIP transcription factor domain in its cytosolic amino terminus. When unfolded proteins accumulate in the ER, ATF6 transits to the Golgi compartment, where it is cleaved by site-1 protease (S1P) and site-2 protease (S2P) to produce a p50 cytoplasmic soluble bZIP-containing transcription factor [12,14,18,20]. In mammals, there are two homologs of ATF6, ATF6α and ATF6β [21,22]. BiP induction is completely abolished in cells that lack S2P and cannot process ATF6 [18], supporting the idea that the primary targets of ATF6 are protein chaperones that augment ER protein-folding capacity. In addition, forced expression of cleaved ATF6α (p50) induced genes encoding ER-resident protein chaperones [23]. However, reduction in *ATF6α* and/or *ATFβ* mRNA did not significantly affect UPR gene induction as analyzed by gene profiling [24]. Thus, the specific roles of ATF6 in activation of ER stress-induced gene expression are in question.

Although three UPR-signal-transducing pathways have been characterized in mammals, it is not known how they coordinate downstream transcriptional activation of different target genes to mediate responses that direct either adaptation or apoptosis when protein folding in the ER is compromised. Unfortunately, the absence of homologous ATF6 and PERK signaling pathways in yeast limits the applicability of studying the UPR in yeast to understanding this process in higher eukaryotes. On the other hand, analysis in mice is complicated by the presence of multiple homologs for IRE1 and ATF6, and the embryonic lethality of homozygous mutations in mammals. Furthermore, studies in MEFs cannot elucidate the physiological and developmental functions of these pathways, because different cell types have different requirements for the UPR sub-pathways [25]. Thus, we selected the nematode *Caenorhabditis elegans* as a model for studying the UPR in mammals. Although a previous cDNA microarray study in *C. elegans* identified only 26 genes that require *xbp-1* for up-regulation in adult nematodes following ER stress [26], we suspected that many more target genes exist. In this study, we used genetic and microarray studies in *C. elegans* to identify the cellular functions of individual UPR signaling pathways and elucidate how these pathways are coordinated during ER stress.

## Results

### *C. elegans atf-6* Complements the *ire-1/xbp-1* Pathway

**Figure 1:**
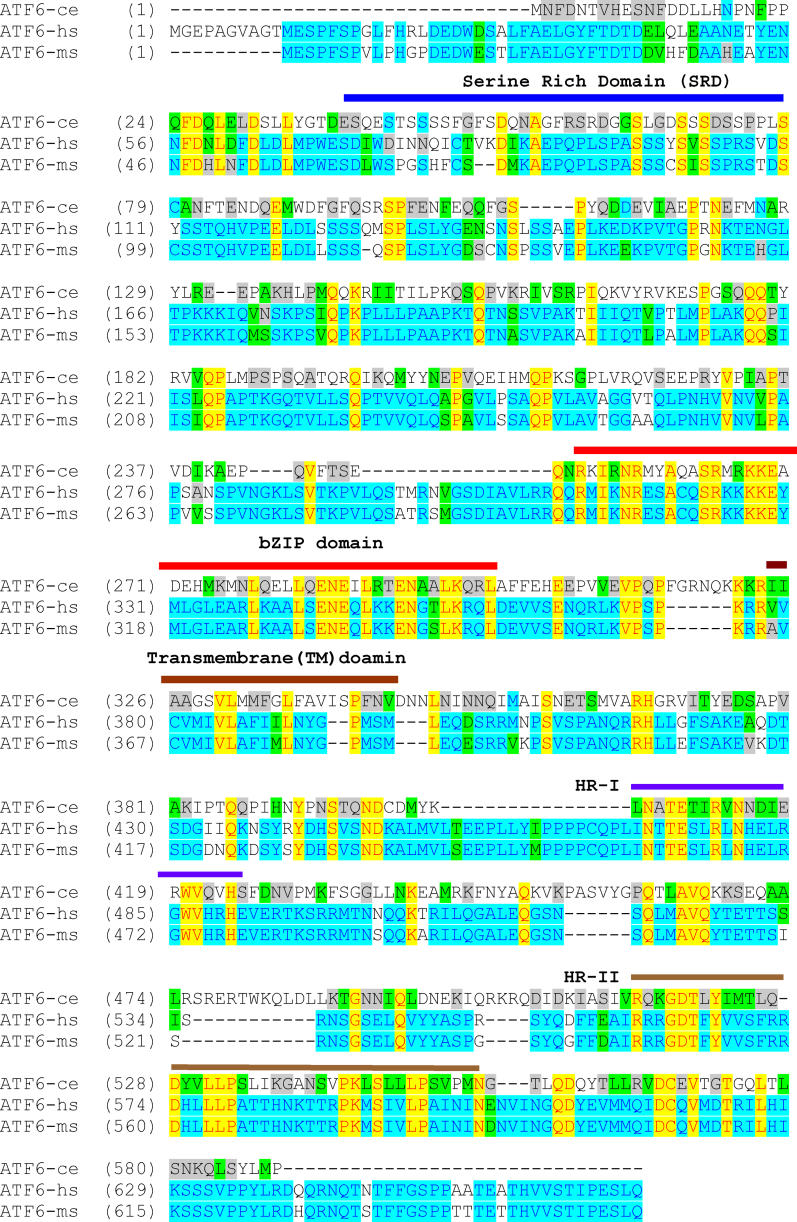
The Sequence Alignment of *C. elegans*, Human, and Murine ATF6 Homologs The sequence alignment of *C. elegans* (ce), human (hs), and mouse (ms) shows five conserved regions in worm ATF-6 including a serine-rich domain, a bZIP domain, a transmembrane domain, and two C-terminal homology regions (HR-I and HR-II). Blue indicates residues that are conservative across species, green indicates blocks of similar residues, yellow indicates identical residues, and grey indicates weak similarity.

Based on sequence homology, we identified F45E6.2 as the only *C. elegans* homolog of *ATF6α,* and named it *atf-6*. *C. elegans* ATF-6 has 22% identity to human ATF6α (Figure 1) and is remotely homologous to human ATF6β (CREBL1, about 16% identity). Worm ATF-6 contains a serine-rich region at the N-terminus, a bZIP domain, and a hydrophobic stretch consisting of 22 residues that is likely to form a transmembrane domain (Figure 1). The C-terminus contains two regions with high homology to their mammalian counterparts that may be required for BiP association and translocation to the Golgi [27]. These similarities suggest that *C. elegans* ATF-6 is a type II ER transmembrane protein that may function like mammalian ATF6α.

**Figure 2:**
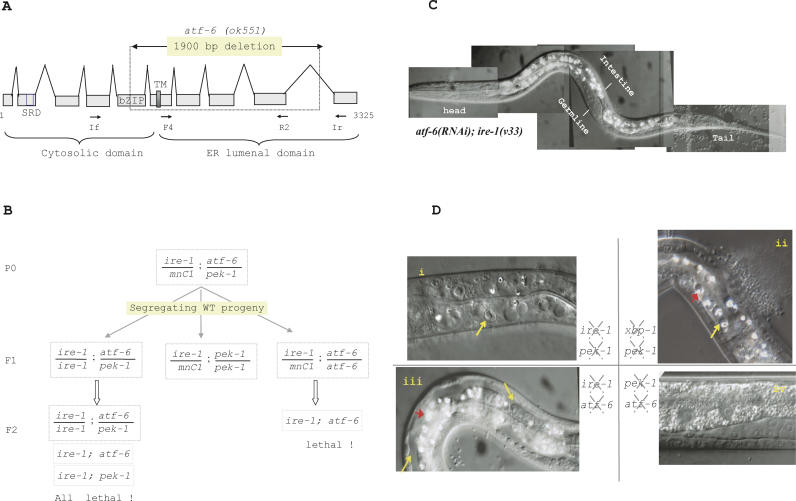
*C. elegans atf-6* and *pek-1* Display Partially Redundant Roles in Complementing *ire-1/xbp-1* for Larval Survival and Development (A) Characterization of *C. elegans atf-6* and its *ok551* deletion allele. The *atf-6* gene structure is depicted in boxes and lines, representing exons and introns, respectively. The *atf-6(ok551)* allele lacks 1,900 bp of genomic sequence, and has the potential to encode a protein without the leucine zipper portion of the bZIP domain, the transmembrane domain, and ER lumenal domain. The *atf-6(ok551)* deletion allele can be detected by PCR, using the primers indicated by arrows. (B) Genetic interactions of *atf-6, ire-1,* and *pek-1*. Animals with the genotype *ire-1(v33); atf-6(ok551)* arrested as young larvae, showing that loss of both *ire-1* and *atf-6* is lethal. The *ire-1(v33)/ mnC1; atf-6(ok551)/ pek-1(ok275)* animals (P0) segregated healthy F1 progeny with the genotype *ire-1(v33); atf-6(ok551)/ pek-1(ok275),* which in turn produced dead F2 animals with exactly the same genotype, suggesting that ATF-6 and PEK-1 function synergistically to cope with endogenous ER stress during development. (C) Nomarski micrograph of a 3-d-old *atf-6(RNAi); ire-1(v33)* animal. The germline of this animal did not develop past the L2 larval stage. (D) Comparisons of intestinal degeneration in various double mutants: (i) *ire-1(v33); pek-1(ok275),* (ii) *xbp-1(RNAi); pek-1(ok275),* (iii) *ire-1(v33); atf-6(RNAi),* and (iv) *atf-6(RNAi); pek-1(ok275*). Normaski micrographs show a portion of the intestine. Mutants in (i)–(iii) arrested at the L2 larval stage and showed intestinal degeneration. The mutants in (iv) had an intestinal morphology similar to the wild-type. Yellow arrows indicate vacuoles in intestinal cells. Red arrowheads indicate light-reflective aggregates appearing in some mutants ([ii]and [iii]).

To elucidate the function of *atf-6,* RNA interference (RNAi) was used to inactivate the gene. The *atf-6(RNAi)* animals had no obvious phenotype differences from wild-type. However, *atf-6(RNAi); ire-1(v33)* double mutants were sluggish and sick, arrested development at the L2 larval stage, and died soon thereafter. They showed intestinal degeneration similar to that found in *ire-1; pek-1* double mutants (Figure 2), and developed many vacuoles in the intestinal cells. As expected, *atf-6(RNAi); xbp-1(RNAi)* animals also died early in larval development. Interestingly, RNAi-mediated silencing of *C. elegans* S2P together with *ire-1* also caused L2 arrest and intestinal degeneration, although silencing S2P alone caused no phenotype abnormalities (data not shown). These results show that either signaling through ATF-6/S2P or through IRE-1/XBP-1 is sufficient for normal development. Furthermore, the findings imply that *C. elegans* ATF-6 may be regulated by S2P-mediated proteolytic cleavage as in mammals. Since *C. elegans* does not have an identifiable S1P homolog, S2P might be the only protease that cleaves ATF-6. Alternatively, it is possible that a protease that is not similar to S1P cleaves ATF-6 prior to cleavage by S2P. For example, in *Escherichia coli* the transmembrane protein RseA is cleaved sequentially by DegS and RseP, and although RseP is a homolog of S2P, DegS is not similar to S1P [28]. Finally, silencing both *atf-6* and *pek-1* caused no obvious phenotype abnormalities (Figure 2D), suggesting that *atf-6* and *pek-1* may function in the same pathway, and that this pathway is partially redundant to the *ire-1/xbp-1* pathway.

### *atf-6* and *pek-1* Share a Common Regulatory Function

To confirm these RNAi results, we characterized the *atf-6* allele *ok551.* DNA sequence analysis revealed a 1,900-bp deletion, extending from 1,276 bp to 3,175 bp downstream of the start codon (Figure 2A). The mutant transcript contains only the first four exons and part of exon 5, and encodes a short protein consisting of the N-terminal transcriptional activation domain and the basic region of the bZIP domain. The leucine zipper, the transmembrane domain, and the C-terminal ER luminal domain are deleted. Because the mutant protein can neither sense ER stress nor bind to its DNA targets, we suspect that it causes a loss of function. Despite this deficit, *atf-6(ok551)* animals appear wild-type, and respond normally to tunicamycin, an agent that induces ER stress by inhibition of asparagine-linked glycosylation (Figure S1).

To dissect genetic interactions among the three known ER stress transducers in *C. elegans,* we constructed the strain *ire-1(v33) II/ mnC1; atf-6(ok551) +/ + pek-1(ok275) X*. This trans-heterozygote for *atf-6* and *pek-1* is stable, since these genes are located near each other on the X chromosome, and the *ire-1(v33)* deletion is balanced by the marker chromosome *mnC1* (Figure 2B). Next, we used PCR genotyping to isolate and study offspring of *ire-1(v33)/ mnC1; atf-6(ok551)* animals. From these heterozygous parents, we found that 0/50 adult offspring had the genotype *ire-1(v33); atf-6(ok551),* but 28/100 eggs were homozygous for both genes*.* We conclude that the *ire-1; atf-6* double mutants die before adulthood, just as we observed using RNAi for *atf-6*.

Animals with the genotype *ire-1(v33)/ mnC1; atf-6(ok551) +/ + pek-1(ok275)* segregated viable *ire-1(v33); atf-6(ok551) +/ + pek-1(ok275)* F1 progeny, but these animals produced F2 progeny that all arrested early in larval development (Figure 2B). The lethality of the *ire-1(v33); atf-6(ok551)* genotype was expected based on our RNAi experiments and the lethality of *ire-1(v33); pek-1(ok275)* was reported previously [7]. However, the finding that *ire-1(v33); atf-6(ok551) +/ + pek-1(ok275)* heterozygotes died, whereas their parents (which had the same genotype) lived, shows that *ire-1* has a maternal effect. This result suggests that *ire-1* might act during embryogenesis or early development. More importantly, these results also show that the loss of a single copy each of the *atf-6* and *pek-1* genes was sufficient to kill *ire-1*-null animals. The observation that haplo-insufficiency for both *atf-6* and *pek-1* is equivalent to loss of both copies of *atf-6,* or both copies of *pek-1,* suggests that *atf-6* and *pek-1* share a common regulatory function.

### IRE-1 Acts through XBP-1 to Induce Transcription of Many UPR Genes

Genetic interactions suggested that *ire-1, pek-1,* and *atf-6* regulate the worm UPR and are required for growth and survival. To elucidate their functions, we performed microarray analysis. Defects in *ire-1/xbp-1* signaling in the presence of a mutation in either *pek-1* or *atf-6* caused L2 larval arrest, implying that UPR signaling may be particularly important at this stage of development. Thus, we carried out a series of microarray analyses using synchronized L2 larvae.

Although the *atf-6(ok551)* allele is synthetic lethal with an *ire-1* deletion, the mutant protein encoded by *atf-6(ok551)* could associate with other DNA-binding proteins to activate transcription. Because RNAi appeared effective at knocking down *atf-6* function, we used *atf-6(RNAi)* animals for the microarray study. To confirm the efficacy of our RNAi treatment, we silenced *atf-6* in *ire-1(v33)* control animals, and found that all *ire-1(v33); atf-6(RNAi)* mutants arrested and died at the L2 stage, which indicates that *atf-6* was efficiently silenced. All synchronized L2 animals, including N2, *ire-1(v33), xbp-1(zc12), pek-1(ok275),* and *atf-6(RNAi),* were treated for 4 h in the absence or 4 h in the presence of 30 μg/ml of tunicamycin to induce ER stress [7,9]. To achieve statistical significance, we prepared RNA samples from independently treated animals for each chip analysis. We performed three biological repeats for each strain and treatment except for tunicamycin-treated *xbp-1(zc12)* animals, for which the analyses were only repeated twice.

**Figure 3:**
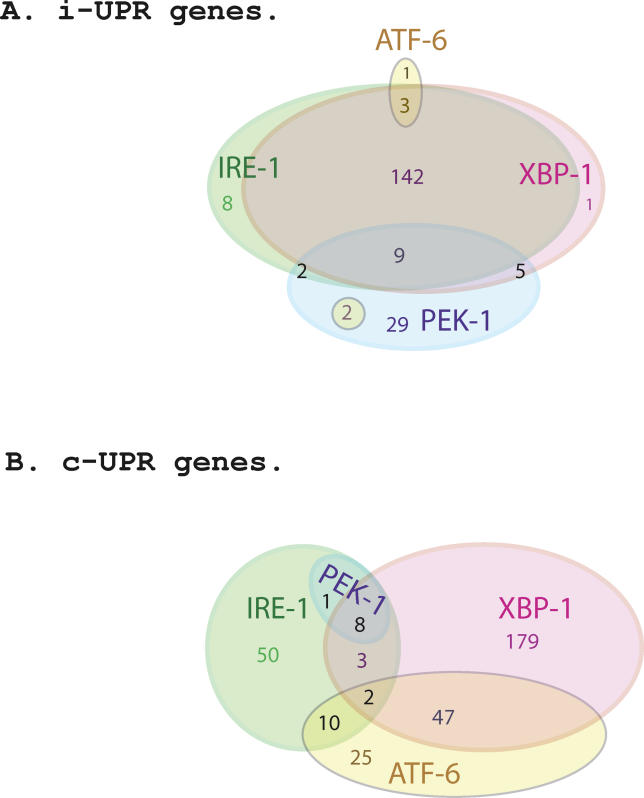
Transcriptional Targets of *ire-1*, *xbp-1*, *pek-1,* and *atf-6* (A) Venn diagram showing the sets of i-UPR genes regulated by *ire-1, xbp-1, pek-1,* and *atf-6*. (B) Venn diagram showing the sets of c-UPR genes regulated by *ire-1, xbp-1, pek-1,* and *atf-6.*

To determine how tunicamycin-induced ER stress affects gene expression in each strain, we used analysis of variance (ANOVA) to study the interactions between each nematode strain and drug treatment [29,30], and identified 4,050 probes with an interaction *p*-value less than 0.01 (one gene could have several probes in the Affymetrix *C. elegans* genome array). This threshold was chosen arbitrarily to ensure that the ANOVA was stringent but would not miss significant genes. Among these 4,050 probes, 202 genes were up-regulated at least 2-fold in wild-type N2 animals by tunicamycin treatment, but not were properly induced in at least one of the mutant strains (i.e., the fold-induction in mutant strains was less than half that observed in the N2 strain). We call this set of 202 genes inducible UPR (i-UPR) genes (Figure 3). The list of 202 genes may underrepresent genes regulated by the i-UPR because of our stringent set of criteria. For example, *cnx-1,* which encodes calnexin, and *crt-1,* which encodes calreticulin, were up-regulated about 1.45-fold and 1.74-fold, respectively, by tunicamycin in an *ire-1/xbp-1*-dependent manner. However, they were not included in the list of i-UPR genes because they had less than 2-fold induction. To confirm the array results, we analyzed 30 genes by quantitative real-time RT-PCR (Figure S2). Expression patterns consistent with the array data were observed for all 30 genes, showing the sensitivity, quality, and validity of our array data.

**Figure 4:**
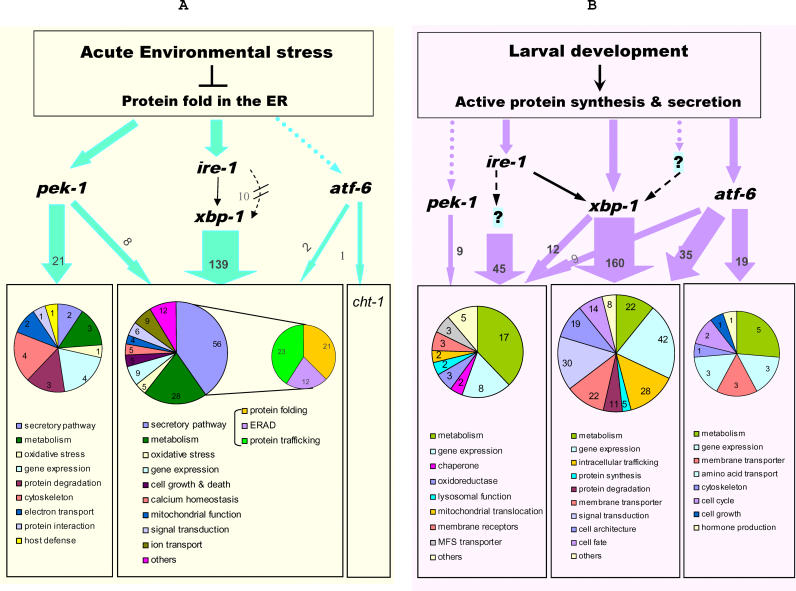
Complex UPR Transcriptional Regulation of Genes with Known Functions in *C. elegans* (A) The i-UPR pathway. Many conditions such as exogenous drug treatment, nutrient deprivation, viral infection, or protein overexpression block or overwhelm protein-folding reactions in the ER and result in ER stress. In this study, we used tunicamycin to block protein folding so as to activate the UPR. Following ER stress, *ire-1* and *xbp-1* act in a linear pathway that dominates the transcriptional response (total of 139 target genes with known functions), inducing genes that reshape the secretory pathway, adjust the metabolic profile, up-regulate functions involved in calcium homeostasis and anti-oxidative stress, and regulate other genes that might affect cell fate. Interestingly, ten genes require either *ire-1* or *xbp-1,* but not both, for their induction upon ER stress. About 21 genes require only *pek-1* for maximal induction, and eight genes regulated by *ire-1/xbp-1* also share regulation by *pek-1*. Finally, *atf-6* does not play a significant role in the i-UPR pathway (depicted by broken arrow). In addition to two genes that were also regulated by *ire-1/xbp-1,* the only gene that depends solely on *atf-6* for its induction is *cht-1,* which encodes a chitinase orthologous to human chitotriosidase. (B) The c-UPR pathway. During development, active protein synthesis and secretion require the UPR signaling molecules *ire-1, xbp-1,* and *atf-6* to maintain the expression of c-UPR genes, defined by the fact that they are not up-regulated by tunicamycin but are dependent on ER stress transducers for expression. Among the genes with known functions, there are only 12 that overlap between the set of 45 genes regulated by *ire-1* and the set of 160 genes regulated by *xbp-1*. In addition, *atf-6* is required by nine genes that are regulated by *ire-1* and 35 that are genes regulated by *xbp-1*. Moreover, the expression of 19 genes is solely dependent on *atf-6,* suggesting an important role of *atf-6* in the c-UPR pathway. By contrast, *pek-1* is largely dispensable for regulation of the c-UPR as only nine genes require *pek-1* in addition to their requirements for *ire-1* to maintain basal expression.

Gene functions were obtained by comparing direct downloads from the Affymetrix Web site to annotations in Wormbase [31]. For uncharacterized worm genes, functions were inferred based on their mammalian or yeast homologs. About 84% of i-UPR genes (170 out of 202 genes) were regulated by both *ire-1* and *xbp-1* (Figure 3A; Table S1). The finding that most *ire-1* and *xbp-1* targets overlap supports the hypothesis that *ire-1* and *xbp-1* function in a single linear pathway. Of genes with known functions, about 40% were involved in the secretory pathway (Figure 4A). *ire-1/xbp-1* was required for tunicamycin-mediated induction of previously characterized protein-folding catalysts including BiP (HSP-4, HSP-3), protein disulfide isomerase (PDI-1, PDI-2), DNJ-7 (also known as PERK inhibitor [p58^IPK^]), and ERO-1, which eliminates highly reactive oxygen species in the ER [6,7,12,17,24,26,32–34], as well as five signal peptidases that would facilitate protein processing under ER stress (Table S1). Previous studies have suggested that the UPR and ERAD are intimately coordinated and UPR induction increases ERAD capacity [6,12]. In addition to previously identified ERAD genes DER-1 (Derlin-1), HRD1, SEL-1 (HRD3), EDEM, and ERD-2 [6,12,18,24,26,35], Derlin-1-interacting AAA ATPase p97, vesicle-fusing ATPase NSF-1, and ER retention protein Rer1 were induced by the UPR in an *ire-1-* and *xbp-1-*dependent manner. Moreover, a set of genes encoding functions involved in various aspects of protein trafficking such as clathrin (mainly COP-II) vesicle formation, translocon assembly, vesicle docking, and signaling events regulating the above processes were part of the i-UPR. The gene *apm-1* (associated protein complex medium chain-1) encodes the μ1 medium chain of the AP-1 clathrin-associated protein complex located at the trans-Golgi complex [36]. Loss of either *ire-1* or *xbp-1* reduced induction of *apm-1,* suggesting that the UPR can also regulate Golgi protein trafficking distal to the ER. Interestingly, *apm-1* RNAi animals arrested at the L1 larval stage with an abnormal intestine [36], a phenotype similar to that observed in *ire-1; pek-1* worms, indicating the importance of the UPR in maintaining organ integrity and function to ensure proper development.

The second largest subset of i-UPR genes (~ 21%) were those involved in lipid, phospholipid, and sugar metabolism, suggesting that the ER couples its protein-folding status with membrane biogenesis and energy consumption and supply (Table S1; Figure 4A). In addition, the i-UPR up-regulated genes that encode functions directly involved in DNA binding and mRNA processing, transport, and translation. Finally, i-UPR genes regulated by *ire-1/xbp-1* were also involved in other processes, such as cell proliferation, calcium homeostasis and intracellular signaling, ion transport, and mitochondrial function (Table S1; Figure 4A).

### The Functions of UPR Genes Regulated by *pek-1* and *atf-6* Complement Those Regulated by *ire-1/xbp-1*

Although previous studies did not detect a set of genes in *C. elegans* regulated by *pek-1,* our array data show that approximately 23% (47 out of 202) of i-UPR genes require *pek-1* for maximal induction (see Figure 3A; Table S2). This percentage is similar to that observed in mammals [16,17], suggesting that *pek-1* plays a similar role in transcriptional regulation of the UPR. The expression of a gene (T04C10.4) homologous to mammalian *ATF4* did not change upon tunicamycin treatment regardless of strain type, consistent with the observation in mammals that *ATF4* expression is not regulated at the transcriptional level (Table S3–S6). i-UPR genes regulated by *pek-1* were involved in various aspects of cell function including the secretory pathway, protein degradation, oxidative stress, metabolism, ion transport, gene expression, and cytoskeleton function (Figure 4A). However, the percentage of secretory pathway genes that required *pek-1* was significantly lower than those that required *ire-1/xbp-1*. In addition, *pek-1* appeared dispensable for the induction of genes directly involved in ER protein folding and ERAD. This result is consistent with our previous observation that *pek-1* is not required for BiP (*hsp-3* and *hsp-4*) induction [7]. Interestingly, 11 genes down-regulated in *pek-1* mutants were up-regulated in *ire-1* and *xbp-1* mutants; five were up-regulated in all of the *ire-1, xbp-1,* and *atf-6* mutants; two were up-regulated in *ire-1* mutants; and one was up-regulated in both *xbp-1* and *atf-6* mutants (Table S2).

Intriguingly, *atf-6* deficiency only affected about six i-UPR genes, none of which appeared to be directly involved in ER protein folding, secretion, or ERAD (Table S2). The negligible role of worm *atf-6* in regulating i-UPR genes involved in the secretory pathway is unexpected based on the proposal that ATF6 helps to regulate protein folding in mammals [18,23]. However, it is consistent with a report showing that a reduction in ATF6α and/or ATF6β does not affect UPR induction in MEFs [24]. The only gene that required *atf-6* was *cht-1,* which encodes a chitinase ortholog of human chitotriosidase, an enzyme belonging to the family of glycosylhydrolases that is massively expressed by lipid-laden macrophages in different lipid-storage diseases including atherosclerosis and Gaucher disease [37,38]. However, the molecular mechanism that underlies the tightly controlled expression of chitotriosidase and how chitotriosidase plays a role in accumulation of lipid material in the lysosomal apparatus is not yet known. One physiological role of human chitotriosidase is likely in innate immunity toward chitin-containing pathogens. In *C. elegans,* CHT-1 may play a role in embryogenesis, and may also be required for cuticle degradation during molting and degradation of chitin-containing pathogens as part of a host defense mechanism [39].

### Constitutive UPR Genes Reveal Physiological Roles for *ire-1, xbp-1, atf-6,* and *pek-1*

During cell growth, differentiation, or physiological responses, there might be constant low-level stress in the ER that requires a basal UPR. Identification of genes that were differentially expressed in *ire-1, xbp-1, pek-1,* and *atf-6* mutants independent of tunicamycin induction might identify normal physiological functions of the UPR. To detect genes that were differentially expressed in mutant worms, we analyzed 8,117 probes (genes) with an *F*-value-associated *p*-value less than 0.001 in the type analysis. Approximately 576 probes (genes) had an average expression that varied more than 2-fold in at least one of the knockout strains compared to wild-type animals (*p* ≤ 0.005). Approximately 228 probes (genes) were up-regulated in at least one of the mutant strains (Table S7), suggesting inhibitory roles of these UPR transducers in regulating these genes during development. In contrast, 324 genes were down-regulated in at least one of the mutant strains, suggesting a requirement for the UPR transducers to maintain their expression. Since expression of these 324 genes was tunicamycin-insensitive but was dependent on one or more of the UPR transducers, we called them constitutive UPR (c-UPR) genes (see Figure 3B). Only one gene (WB protein ID: CE20477) in this list was induced more than 2-fold by tunicamycin (Table S8). However, since all mutants displayed a similar 2-fold induction, CE20477 was classified as a c-UPR gene.

Out of 324 c-UPR genes, the expression of 72 required *ire-1* and 239 required *xbp-1* (Tables S8 and S9). Interestingly, there were only 13 overlapping genes that required both *ire-1* and *xbp-1,* suggesting that *ire-1* and *xbp-1* have additional divergent functions that are separate from the classic i-UPR. In fact, about 816 genes were differentially expressed (>2-fold) in *ire-1* and *xbp-1* mutants (*p* ≤ 0.005; Table S10). Although *C. elegans atf-6* appeared dispensable for the transcriptional regulation of i-UPR genes, it was required to maintain the expression of 26% of the c-UPR genes (84 out of 324 genes), suggesting the importance of *atf-6* in normal cell processes and/or development. Finally, *pek-1* regulated only nine c-UPR genes, and all of them were also dependent on *ire-1* and/or *xbp-1* (Table S9; Figure 4B). c-UPR genes encode proteins involved in a wide range of cellular functions including metabolism, gene expression, protein synthesis and degradation, intracellular trafficking, membrane transport, cytoskeleton function, cell cycle, apoptosis, and signal transduction.

### *CREBh* is a Novel UPR Gene Dependent on *ire-1*, *xbp-1,* and *atf-6*

**Figure 5:**
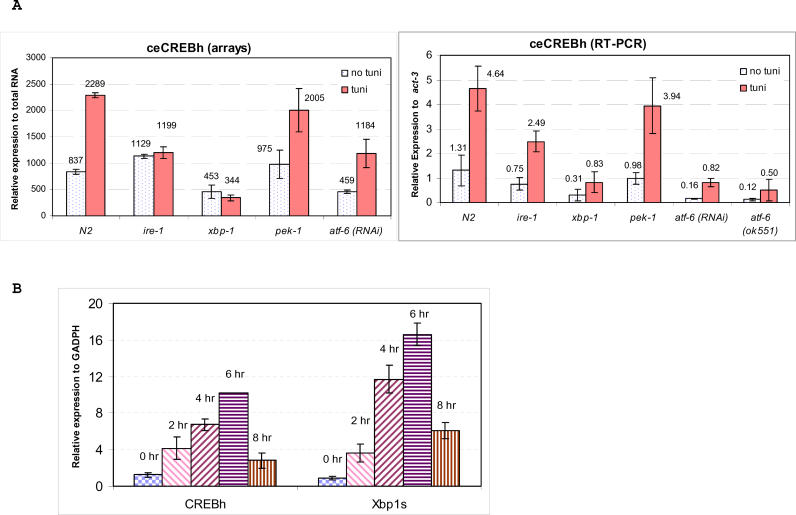
CREBh Is a Novel UPR-Responsive Gene (A) Microarray and quantitative RT-PCR analyses show that the expression of *C. elegans* (ce) CREBh requires *ire-1, xbp-1,* and *atf-6*. “Tuni” indicates tunicamycin treatment, as described in the Materials and Methods section. (B) ER stress induced by dithiothreitol in HepG2 cells activates CREBh transcription. HepG2 cells were treated with dithiothreitol and harvested at various time points from 0 h to 8 h. The relative expression of CREBh and spliced *xbp-1* transcripts (Xbp1s) was analyzed by quantitative RT-PCR and normalized to GADPH. The induction pattern of CREBh resembles that of spliced *xbp-1* transcripts.

In the i-UPR gene list, we identified a gene—F57B10.1—encoding a bZIP transcription factor homologous to mammalian *CREBh*. *C. elegans CREBh* was up-regulated about 2.73-fold upon tunicamycin treatment in N2 worms. However, this up-regulation was abolished in *ire-1* and *xbp-1* mutant worms (Figure 5A). Although *atf-6(RNAi)* worms showed 2.6-fold induction of *CREBh,* both basal and stimulated levels were significantly reduced. In contrast, *pek-1* was not required for *CREBh* expression. To confirm the requirement of *ire-1*/*xbp-1* and *atf-6* in *CREBh* expression, we performed real-time quantitative RT-PCR analysis (Figure 5A). Both the basal and stimulated expression levels of *CREBh* in *ire-1, xbp-1,* and *atf-6* mutants were significantly reduced. While *ire-1* mutants showed one-half the expression of wild-type animals, *xbp-1* and *atf-6* mutants showed more dramatic reductions in *CREBh—*about 4.2- and 10-fold, respectively. The small differences in *CREBh* expression between microarray and real-time PCR analysis were probably due to different normalization methods. In the microarray analysis, gene expression was normalized to the total hybridization intensity based on the assumption that the total amount of RNA per cell does not change with different conditions. In the RT-PCR analysis, gene expression was normalized to the expression of *act-3,* a house-keeping actin gene. Nevertheless, both sets of data show that *C. elegans CREBh* is a novel gene regulated by both *ire-1*/*xbp-1* and *atf-6.* Both *atf-6(RNAi)* and *atf-6(ok551)* worms showed very similar *CREBh* expression patterns, supporting the hypothesis that *ok551* is a loss-of-function allele and that *atf-6* regulates *CREBh* expression.

To determine whether mammalian *CREBh* also responds to ER stress, we analyzed *CREBh* expression in a human hepatoma cell line—HepG2, upon ER stress induced by dithiothreitol, which blocks protein folding by interfering with disulfide-bond formation. *CREBh* transcripts were induced and peaked at 6 h following dithiothreitol treatment with ~8.7-fold up-regulation, and then sharply declined at 8 h (Figure 5B). The transient induction pattern of *CREBh* mimicked that of spliced *xbp-1,* confirming that *CREBh* is a UPR-responsive gene in both *C. elegans* and mammals.

## Discussion

### The *ire-1/xbp-1* Pathway Controls Most i-UPR Genes in Worms

In yeast, the UPR is solely dependent on Ire1p and its splicing target *HAC1*. By contrast, mammalian IRE1/XBP1 appears to be required primarily for the induction of genes involved in ERAD, based on the finding that the expression and induction of BiP but not EDEM is intact in *ire1*-deficient MEFs. We found that *C. elegans ire-1/xbp-1* regulates the majority (~ 83%) of the classic i-UPR genes, including genes functioning in both protein folding and ERAD. This result suggests that worm *ire-1* and *xbp-1* have broader functions in the UPR than their mammalian homologs, and implies that the UPR of *C. elegans* strongly resembles that of yeast.

Homozygous mutations in either *pek-1* or *atf-6*, or haplo-insufficiency for both *pek-1* and *atf-6* failed to complement *ire-1*-null mutants. By contrast, *pek-1(RNAi); atf-6(RNAi)* double mutants were normal. This result implies that the *ire-1* pathway plays a more important role in the worm UPR and development than either *pek-1* or *atf-6*. This notion is supported by our discovery that *ire-1* and *xbp-1* control the expression of the vast majority of both i-UPR and c-UPR genes. The broader role of *ire-1/xbp-1* for the UPR in *C. elegans* compared to mammals is not surprising since worms are developmentally simpler than mammals. During evolution, mammalian genes gained more diverse, specific, as well as fine-tuned regulation. For example, both mammalian IRE1 and PERK are selectively critical for specific developmental programs and functions. Disruption of either pathway causes lethality and growth defects associated with hypoplasia or malfunctions of selective secretory organs, such as hepatocyte and B lymphocyte defects in *ire1* ^−/−^ mice and pancreatic beta cell defects in *perk* ^−/−^ mice [16,19,25,40–42].

### The UPR Is Controlled by Complex Genetic Interactions and Is Essential for Development

The requirement for PERK and ATF6 homologs in both *C. elegans* and mammals indicates that they both differ from yeast, perhaps because metazoans normally experience ER stress during differentiation of highly specialized cells and tissues. Generating a complex set of differentiated tissues might require multiple ER sensors that detect and respond to a variety of demands placed on the ER during cell differentiation. This hypothesis is supported in *C. elegans* by the finding that double mutants defective in either *ire-1/xbp-1* and *pek-1* or *ire-1/xbp-1* and *atf-6* die specifically as L2 larvae with intestinal degeneration.

A few i-UPR and c-UPR genes were up-regulated in some UPR gene mutants but were down-regulated in others. These genes are highlighted in purple in tables. This dichotomous regulation is most apparent for i-UPR genes regulated by *pek-1,* as shown in Table S2. One possible explanation is that the loss of *ire-1, xbp-1,* or *atf-6* induces ER stress, which up-regulates *pek-1* signaling. Alternatively, *ire-1/xbp-1* and *atf-6* could inhibit the expression of *pek-1*-dependent genes. Currently, we cannot distinguish between these two possibilities. The synthetic lethality observed in either *ire-1; pek-1* double mutants or *ire-1; atf-6* mutants may result from both compensatory gene regulation among the *ire-1/xbp-1*, *pek-1,* and *atf-6* pathways and/or functional complementation between the i-UPR and c-UPR pathways.

### IRE-1 and XBP-1 Regulate Different Subsets of c-UPR Genes

In *C. elegans,* most i-UPR genes controlled by *ire-1* also require *xbp-1* (see Figure 4A; Table S1). This observation isn't surprising, since these genes are thought to act in a linear pathway, in which IRE-1 mediates the splicing of *xbp-1* mRNA to create a potent bZIP transcription factor. There are only ten exceptions to this rule among i-UPR genes; examples include *arf-1.1,* which requires only *xbp-1,* and T12D8.5, which requires only *ire-1.* By contrast, almost all c-UPR genes require either *ire-1* or *xbp-1,* but not both (Figure 4B; Tables S8 and S9). In addition, *C. elegans xbp-1; pek-1* double mutants display many reflective crystal-like aggregates in their degenerating intestines, whereas these are absent in *ire-1; pek-1* double mutants (see Figure 2D). This phenotypic difference supports the idea that *ire-1* and *xbp-1* functions don't completely overlap, especially since these differences are found in animals that were raised under non-UPR-inducing conditions.

Since *xbp-1* mRNA does not appear to be spliced under physiological conditions [7], there is no reason why *ire-1* and *xbp-1* should act on the same target genes. What is surprising is that *xbp-1* functions at all. IRE-1 could regulate other target genes as a kinase, or by controlling their splicing. However, it is unlikely that worm *xbp-1* is cleaved by something other than *ire-1,* since spliced *xbp-1* mRNA is not detected in *ire-1* mutants [7]. Instead, unspliced *xbp-1* mRNA must function independently of IRE-1. Although this doesn't occur in yeast, metazoan *xbp-1* is not an exact homolog of yeast *HAC1*. Not only do they share minimal amino acid sequence homology, but unspliced *HAC1* mRNA is translationally attenuated, whereas unspliced *xbp-1* is not [9,43]. Furthermore, unspliced *xbp-1* encodes a transcription factor that can act as a dominant-negative regulator of spliced XBP-1 [40,44].

Although *ire-1* and *xbp-1* regulate genes that function in similar processes, such as metabolism and gene expression, the depth and breadth of the regulation were shown to be different (see Figure 4B; Tables S8 and S9). For example, *ire-1* regulated only eight genes involved in gene expression, while *xbp-1* regulated 42 genes that function in many different aspects of gene regulation. Fifteen are transcription factors, and the others act in diverse processes ranging from RNA synthesis, processing, and export to RNA catabolism; from transcription to translation; and from DNA repair to gene silencing. In addition, many genes involved in protein turnover, intracellular trafficking, membrane transport, cell fate decisions, signal transduction, cytoskeletal structure, neuronal functions, etc., were dependent on *xbp-1* but not on *ire-1* (Table S9). In total, the expression of 816 genes depended on either *ire-1* or *xbp-1,* but not both (Table S10).

Our findings are supported by recent evidence that murine IRE1α plays multiple roles in both the early and the late stages of B cell development, while XBP1 is only required for the terminal differentiation of B cells into plasma cells [25,40,41]. In the early stages, IRE1α regulates c-UPR genes (such as *TDT [terminal deoxynucleotidyl transferase]* and the recombination-activating genes *RAG1* and *RAG2*), and is required for immunoglobulin gene rearrangement and B cell receptor formation. Interestingly, the IRE1α-dependent regulation of *RAG1, RAG2,* and *TDT* does not require either the IRE1α kinase or endoribonuclease activities [25]. During terminal B cell differentiation, the IRE1α endoribonuclease initiates splicing of *XBP1* mRNA to produce the XBP1 transcription factor that is required for plasma cell differentiation and antibody production [40,41].

Furthermore, we found that *ire-1,* but not *xbp-1,* is required for basal expression of C49F5.1, which encodes S-adenosylmethionine synthetase, an enzyme that catalyzes the formation of *S*-adenosylmethionine, the principal biological methyl donor and precursor for the synthesis of adenosine and homocysteine [45]. In the mammalian liver, *S*-adenosylmethionine plays a pivotal role in the regulation of cellular proliferation, differentiation, and apoptosis, and its levels must be tightly controlled [46]. S-adenosylmethionine synthetase expression in fetal liver isolated from *ire1α^−/−^* mouse embryos was approximately 3.5-fold reduced compared to that observed in their wild-type littermates, whereas *xbp1* ^−/−^ embryos showed proper expression of S-adenosylmethionine synthetase (K. Zhang and R. Kaufman, unpublished data). This result implies that S-adenosylmethionine synthetase is a novel IRE1-dependent c-UPR gene.

### *C. elegans* ATF-6 Regulates a Set of Genes That Are Not Inducible by ER Stress

Blast search revealed that the *C. elegans* gene F45E6.2 is most closely related to mammalian ATF6α, with low homology to mammalian ATF6β, so we named it *C. elegans atf-6*. Our data show that *C. elegans atf-6* does not regulate induction of i-UPR genes, but does control approximately one-quarter of c-UPR genes. Therefore, *atf-6, ire-1,* and *xbp-1* are required for the expression of most c-UPR genes, and the *pek-1* and the *ire-1/xbp-1* pathways are required for the expression of most i-UPR genes (see Figure 3). An alternative model is that worm *atf-6* might have evolved as a backup mechanism to the *ire-1/xbp-1* pathway, since many genes regulated by *atf-6* overlap with those regulated by *ire-1* and *xbp-1*.

The worm microarray results showing that *atf-6* does not regulate the i-UPR genes are consistent with the observation that *atf-6(ok551)* mutants and *atf-6(RNAi)* animals are as resistant to tunicamycin as wild-type worms (see Figure S1), whereas both *ire-1* and *pek-1* mutants are hypersensitive to tunicamycin [7]. However, it is possible that *atf-6(RNAi)* animals have residual activity because of incomplete silencing, and that this activity is sufficient to regulate gene expression, even though the *atf-6 (RNAi); ire-1(v33)* double mutant phenotype was lethal. In addition, we found that both the full-length and the N-terminal nuclear forms of worm *atf-6* did not increase the activity of a mammalian ATF6α luciferase reporter in MEFs (data not shown). However, the DNA sequence recognition motif for the *C. elegans* and mammalian ATF6 homologs may have diverged. Alternatively, it is possible that during evolution mammalian ATF6 gained additional functions, such as up-regulating genes encoding proteins that facilitate protein folding in the ER.

The hypothesis that mammalian ATF6 regulates protein folding following ER stress was based on indirect evidence, including (1) the forced expression of ATF6α [23], and (2) analysis of a cell line that lacked S2P and thus failed to process ATF6 and activate BiP gene expression [18]. However, we do not believe that these data are conclusive. First, a reduction in ATF6α and/or ATFβ by RNAi produced minimal effects on UPR gene induction, as monitored by gene profiling [24]. Second, a number of transcription factors exist that are targets of S2P, such as Luman [47], Oasis [48], and CREBh [49,50]. One of these other S2P-dependent ER stress-induced transcription factors might control the induction of UPR target genes, like *BiP*. Our analysis of *C. elegans atf-6* suggests that mammalian ATF6, identified as a serum response factor, may not be a typical UPR transducer like IRE1 and PERK. To firmly establish the function of mammalian ATF6, it will be necessary to analyze ATF6α and ATF6β knockout mice.

As the *C. elegans* intestine is the first organ to encounter many environmental toxins and infectious agents, it is likely that tunicamycin is more accessible to intestinal epithelial cells than to cells of other tissues, such as neurons. Expression analysis using promoter-driven green fluorescent protein (GFP) showed that both *atf-6* and *pek-1* are strongly expressed in the intestine, as well as neurons and muscles (Figure S3). In addition, an *hsp-3* promoter–GFP fusion was expressed at a low level, but was highly inducible by tunicamycin treatment in the majority of the tissues in the worm (Figure S4). Finally, many of the i-UPR and c-UPR genes encode functions for general cell processes that are expressed ubiquitously, such as protein synthesis and degradation, intracellular trafficking, metabolism, and cell cycle. These observations suggest that the i-UPR does not selectively exist in the intestine, and likewise, the c-UPR is not selectively present in non-intestinal tissues.

### Gene Targets of the UPR Regulate Diverse Functions

Our microarray studies identified most of the UPR genes reported previously, including 17 of the 26 *xbp-1-*dependent genes identified in a nematode cDNA array study [26] (Table S11). This cDNA study also identified a family of ten *abu* genes (activated in blocked UPR), that were induced to higher levels in ER-stressed *xbp-1* mutant animals than in ER-stressed wild-type animals [26]. However, we found that none of the *abu* genes were up-regulated in either *ire-1* or *xbp-1* mutant worms (Table S11), perhaps because we studied L2 larvae and most of the *abu* genes appear to be expressed in older worms.

Our array data not only identified additional genes involved in key functions like protein folding, translocation, and ERAD, but also revealed additional cellular processes affected by the UPR. For example, a set of genes proposed to regulate calcium homeostasis is induced upon ER stress in an *ire-1/xbp-1*-dependent manner (Table S1; see Figure S2). This set includes SERCA *(sca-1),* ryanodine receptor *(unc-68)* [51], calumenin [52], nucleobindin [53,54], Herp [55], BAP31 [56], and Ca^2+^-independent phospholipase A2 (iPLA2) [57]. These findings suggest that the cell enhances its overall ability to handle calcium, a cation that is crucial to the delicate balance between cell survival and apoptosis upon ER stress. In addition, the i-UPR up-regulates genes that are directly involved in cell proliferation and differentiation such as CDK5 activator-binding protein C53 and the piwi/argonaute family protein eIF2C4.

Phosphatidylcholine (PC) is a major structural component of cell membranes and an important source for signaling molecules such as diacylglycerol [58]. The de novo synthesis of PC from choline consists of three steps: choline kinase converts choline into phosphocholine, which is then converted into CDP-choline. In the last step, choline/ethanolaninephosphotransferase catalyzes the transfer of phosphocholine from CDP-choline to diacylglycerol. We found two genes (F22F7.5 and B0285.9) encoding choline kinases that were up-regulated by tunicamycin in an *ire-1*/*xbp-1*-dependent manner (Table S1; Figure S2). In addition, *ire-1, xbp-1,* and *atf-6* were all required to maintain expression of choline/ethanolaninephosphotransferase in either a physiological context or upon encountering acute environmental stress. Increased expression and activity of choline kinase were reported to associate with cancer, while decreased PC synthesis induced growth arrest and apoptosis [58,59]. Therefore, up-regulation or maintenance of PC synthesis by both the i-UPR and the c-UPR may provide a survival signal confronting ER stress. Recently, it was proposed that up-regulation of choline phosphotransferase may contribute to the membrane expansion that accompanies activation of the UPR during B lymphocyte differentiation into plasma cells [41,60].

### *CREBh* Is a Novel UPR Gene

Finally, our microarray data identified *CREBh* as a novel UPR gene in both worms and mammals. *C. elegans CREBh* appears to be essential in worm development as suggested by embryonic lethality in *CREBh*-silenced worms [61]. CREBh belongs to the CREB/ATF family of transcription factors and its expression is restricted to the liver [49]. *CREBh* induction during fetal liver development is abolished in *ire1α^−/−^* mouse embryos (K. Zhang and R. Kaufman, unpublished data), suggesting an evolutionary conserved mechanism. However, it is not known whether mammalian XBP1 and/or ATF6 regulate *CREBh* expression. Interestingly, CREBh has a transmembrane domain that strongly resembles that of ATF6. Elucidating whether CREBh is cleaved and activated similar to ATF6 under ER stress and understanding its biological functions will broaden our view of the UPR in liver development and function.

###  Conclusion

In summary, transcription profiling in *C. elegans* revealed two aspects of the UPR: the i-UPR pathway directs cells to respond to acute environmental stress, and the c-UPR pathway is an essential component of the UPR during normal development (see Figure 4). In the i-UPR pathway, *ire-1* and *xbp-1* act in a linear process that dominates transcriptional regulation to reshape the secretory pathway and adjust cellular functions involved in calcium and phospholipid homeostasis, cell proliferation and death, anti-oxidative stress, metabolism, energy generation, cytoskeletal structure, and mitochondrial function. In addition, *pek-1* is necessary for the maximal induction of one-quarter of i-UPR genes, but *atf-6* plays little role in the classic i-UPR since few genes require *atf-6* for their induction upon ER stress. By contrast, *atf-6* plays a more important role than *pek-1* does in the c-UPR pathway. Furthermore, *ire-1* and *xbp-1* regulate very different sets of c-UPR genes, so their normal physiological functions have diverged. This observation implies that alternative regulatory mechanisms can activate or transmit signals downstream of *ire-1* and *xbp-1*. Finally, the UPR regulates genes functioning in a much broader range than those previously reported or expected from analysis of yeast and other model systems, and many of these regulatory interactions seem to be conserved in mammals. We believe that genetic and genomic profiling analysis in worms provides a missing link between the yeast and the mammalian UPRs, and sheds light on the role of the mammalian UPR in development and disease.

## Materials and Methods

### Strains and general methods.

*C. elegans* strains were cultivated at 20 °C [62]. The strain N2 (Bristol) was used as the wild-type. The *atf-6(ok551)* mutant was obtained from the *C. elegans* Gene Knockout Consortium (http://celeganskoconsortium.omrf.org/). The mutant strains *ire-1(v33), pek-1(ok275), xbp-1(zc12),* and *mnC1* were described previously [7,9]. To construct the strain *ire-1(v33)/ mnC1; atf-6(ok551)/ pek-1(ok275),* we crossed *ire-1(v33)/mnC1; pek-1(ok275)* with *atf-6(ok551).* The *ire-1(v33)/ +; atf-6(ok551)/ pek-1(ok275)* genotypes were selected by PCR and crossed to *ire-1(v33)/ mnC1; pek-1(ok275)*. Offspring with the genotype *ire-1(v33)/ mnC1; atf-6(ok551)/ pek-1(ok275)* were identified by PCR genotyping. Genotyping of *ire-1(v33)* and *pek-1(ok275)* was described previously [7].

### Genotyping and characterization of the *atf-6(ok551)* deletion mutant.

The primers *atf-6*-If (5′-AATGACCAGGAAATGTGGGA-3′) and *atf-6*-Ir (5′-AAGTGTCAATTGGCCAGTCCCTGT-3′) were used to detect the *atf-6(ok551)* deletion allele. The wild-type *atf-6* allele amplified a 2,980-bp fragment compared to a 1,100-bp fragment from the *atf-6(ok551)* allele. Homozygous *atf-6(ok551)* mutants were identified by PCR using primers *atf-6*-F4 (5′-CGGAAGAGTCATCACGTATGAAG-3′) and *atf-6*-R2 (5′-GGCAGAAGCACGTAGTCTTGAAG-3′) from inside the deletion region. These two reactions were performed in one PCR tube by mixing all four primers. Wild-type animals generated only one 780-bp fragment amplified by primers F4 and R2. Homozygous *atf-6(ok551)* mutants had only one 1,100-bp fragment amplified by If and Ir, whereas heterozygous mutants had both 1,100-bp and 780-bp fragments.

### RNA interference by injection.

PCR was used to amplify cDNA fragments flanked by the T7 promoter at both the 5′ and 3′ ends. The primer pair T7-5ceATF6 (5′-GGATCCTAATACGACTCACTATAGGAGACGGCGGGAGTTTAGGAGATTC-3′) and T7-3ceATF6 (5′-GGATCCTAATACGACTCACTATAGGCTTGATTTGGCGTTGCGTAGC-3′) amplified an approximately 520-bp fragment encoding the N-terminus of ATF-6. Amplified templates were transcribed in vitro to yield dsRNA [63] for injection as described [64]. Only progeny hatched from eggs laid between 12 and 24 h postinjection were studied. Silencing of *xbp-1* by RNAi was described previously [7].

### RNAi-mediated silencing *atf-6* genes by bacterial feeding.

The primers 5′-TGTTTTAGATCTGGCGGGAGTTTAGGAGATTC-3′ and 5′-AATATGGTACCTTGATTTGGCGTTGCG-3′ were used to amplify a 430-bp cDNA fragment of *atf-6,* which was cloned into the BglII and KpnI sites in the L4440 feeding vector (pPD129.36). The resulting *atf-6* RNAi plasmids were transformed into the HT115 (DE3)—an RNase III–deficient *E. coli* strain [65]. Resistance to ampicillin and tetracycline was used to select transformed cells. A small LB culture containing ampicillin (100 μg/ml) and tetracycline (12.5 μg/ml) was inoculated overnight, and diluted 1:100 into a large LB culture that contained ampicillin (50 μg/ml). When the OD at 600 nm reached 0.6, IPTG was added to a final concentration of 1 mM to allow induction for 4 h. A batch of bacteria expressing *atf-6* dsRNA was centrifuged and stored at 4 °C for further use. Another batch of bacteria was seeded directly onto NMG-Lite plates containing 1 mM IPTG and 50 μg/ml ampicillin. Seeded plates were dried at room temperature and induction was continued at room temperature overnight. To prepare *atf-6(RNAi)* worms for microarray analysis, synchronized L4 larvae (N2 or *ire-1[v33]*) were fed with bacteria expressing *atf-6* dsRNA in liquid culture for 1.5 d and then bleached to produce many eggs, which were then transferred to seeded *atf-6* RNAi plates. After 20 h, the resulting *atf-6(RNAi)* L2 worms were transferred and treated with or without 30 μg/ml tunicamycin for 4 h in liquid culture before harvesting RNA. As a positive control to ensure the efficiency of *atf-6* RNAi, nearly all *ire-1(v33); atf-6(RNAi)* worms died in 2 d on *atf-6(RNAi)* plates and no survivors were observed at 5 d, suggesting that the *atf-6* gene was efficiently silenced.

### RNA isolation and cRNA synthesis for microarray analysis.

Synchronized adult worms including N2, *ire-1(v33), pek-1(ok275),* and *xbp-1(zc12)* animals were bleached to isolate eggs, which were transferred to 150-mm NGM-Lite plates to allow growth for 20 h. All the L2 larvae were then transferred to liquid culture in the presence or absence of 30 μg/ml tunicamycin for 4 h. After treatments, worms were centrifuged and pellets frozen in liquid nitrogen for RNA preparation. Total RNA was isolated by using TriReagent from MRC (Cincinnati, Ohio, United States) and purified using the Qiagen (Valencia, California, United States) RNeasy cleanup procedure. The quality of total RNAs was assessed by Agilent (Palo Alto, California, United States) Bioanalyzer. The cRNAs were synthesized from 10 μg of total RNA by CyScribe kit from Amersham Pharmacia Biotech (Piscataway, New Jersey, United States) and fragmented in the presence of heat and Mg^2+^ before hybridization to the Affymetrix *C. elegans* Genome Genechip, which represents 22,500 transcripts from *C. elegans*.

### Microarray analysis.

Each RNA sample used for cRNA synthesis and chip hybridization was isolated from independently prepared worm samples so that the repeats for each strain and treatment were biologically significant. Although a total of 29 chips were processed in five different batches, RNA digestion plot and density curves ensured that they were all comparable. An ANOVA model (*y_ij_ =* α*_i_ +* β*_j_ +* [αβ]*_ij_ +* ɛ*_ij_*) was fit to the expression values computed from all 29 chips. Here *y* is the expression value, α is the main effect for treatment, β is the main effect for strain type, and αβ is the type-plus-treatment interaction term. In this analysis we were only interested in the interaction and type terms. In the overall model-fit data, 13,706 probes had a *q*-value less than 0.001. Global significance was determined by using permutation tests. On the average of permutation tests, we identified only 27 probes with a *q*-value equal to 0.001, suggesting the significance of our data. Significant genes in overall model-fit data were then filtered into 4,050 probes by their interaction *p*-value being less than 0.01. About 265 genes represented by 307 probes were up-regulated at least 2-fold in the N2 strain upon tunicamycin treatment and 202 of them failed to be induced in at least one of the mutant strains as their folds of induction were less than a half of that observed in the N2 strain.

After filtering out probes significant in the interaction term, we analyzed the type term for the remaining significant genes in overall model-fit data. There were 8,117 probes with a *p*-value associated with *F*-value less than 0.001 when we did cross-comparison between any two strain types. The fold changes were calculated by comparing the average expression of both nontreated and treated mutants to that in N2 strain samples, and the corresponding *p*-value for each comparison was calculated. Among approximately 8,117 significant probes, the expression of about 576 varied at least 2-fold in at least one of the mutant strains compared to the N2 strain and had a corresponding *p*-value less than 0.005. About 324 genes were down-regulated in the mutants and further studied. About 228 probes were up-regulated in the mutants and not discussed in the paper (Table S7).

### Quantitative real-time RT-PCR analysis.

The cDNA was synthesized from 400 ng of total RNA in a 20-μl reaction and then diluted into 800 μl. To detect targeted transcripts, 9 μl of the diluted cDNA was mixed with 1 μl of primer sets (1 μM for each primer) and 10 μl of sybr green mix from Applied Biosystems (Foster City, California, United States). The reactions were run at step 1, 95 °C for 10 min, and step 2, 95 °C for 10 s followed by 60°C for 50 s for 40 cycles on a Bio-Rad (Hercules, California, United States) iCycler. The PCR product was captured at real time during amplification at 60 °C, and Ct numbers were obtained. *C. elegans* gene expression was normalized to the expression of *act-3,* a house-keeping actin [7]. The primer pair 5′-TCAGAACTCAAATGGTCTTGTCAGA-3′ and 5′-ATGACGAAGATGGTGCATTGAG-3′ was used to detect *C. elegans* CREBh. The expression of human genes was normalized to GADPH (Applied Biosystems). The primers 5′-CATCATCCTCCCCTCCATCA-3′ and 5′-GAACACTCGTACAGGCGCAAA-3′ were used to detect human CREBh. The primers 5′-CCGCAGCAGGTGCAGG-3′ and 5′-GAGTCAATACCGCCAGAATCCA-3′ were used to analyze the spliced human *XBP1* transcripts.

## Supporting Information

Figure S1 *atf-6 (ok551)* Mutants Are Resistant to ER Stress Induced by Tunicamycin Gravid adults were allowed to lay eggs for 4 h on plates in column a, and then were transferred to the corresponding plates in column b. After 4 h to permit egg laying, the animals were transferred to plates in column c for another 4 h of egg laying. Eggs from each strain were laid on plates containing either 5 μg/ml tunicamycin (A) or 7.5 μg/ml tunicamycin (B), and after 3 d the numbers of adult worms appearing on plates were counted. The percentages of worms maturing to adulthood were calculated.
(10 KB PDF)

Figure S2 RT-PCR Verification of Microarray Data Thirty genes from the I-UPR gene list were picked randomly and analyzed their expression by quantitative RT-PCR. All gene expression levels were normalized to that of *act-3*.
(72 KB PDF)

Figure S3 Expression Patterns of *C. elegans atf-6* and *pek-1*
(A) *pek-1-*promoted GFP.
(B) *atf-6-*promoted GFP. The upstream 3.5-kb promoter regions of the *pek-1* gene and the *atf-6* gene were fused to the coding region of GFP (Fire lab GFP expression vector ppD95.75). The expression patterns were confirmed in at least five independent stable lines carrying extrachromosomal arrays of the GFP fusion constructs.
(88 KB PDF)

Figure S4 *hsp-3*-Promoted GFP Worms The 2-kb promoter region of the *hsp-3* gene was inserted upstream of GFP and introduced as an extrachromosomal array into *C. elegans*. GFP was analyzed after 36 h following hatching in plates lacking (top) or containing (bottom) of 10 μg/ml tunicamycin.
(17 KB PDF)

Table S1 i-UPR Genes Regulated by *ire-1/xbp-1*
This table summarizes genes that are up-regulated at least 2-fold upon tunicamycin treatment in N2 animals but are not up-regulated in *ire-1* and/or *xbp-1* mutants. Fold changes were obtained by comparing tunicamycin-treated samples with untreated samples. The fold change and mean expression are represented in log(2) algorithm. Significant down-regulations in mutants are highlighted in yellow (*p* < 0.01). Some genes are down-regulated in some mutants but up-regulated (highlighted in purple) in others. Genes marked by an asterisk are essential genes based on knockout mutations or RNAi analysis as listed on the Wormbase Web site.
(76 KB XLS)

Table S2 i-UPR Genes Regulated by *pek-1* and *atf-6*
(A) and (B) summarize genes that are up-regulated at least 2-fold upon tunicamycin treatment in N2 animals but are not up-regulated in *pek-1* (A) or *atf-6* (B) mutants. Fold changes were obtained by comparing tunicamycin-treated samples with untreated samples. The fold change and mean expression are represented in log(2) algorithm. Significant down-regulations in mutants are highlighted in yellow (*p* < 0.01). Some genes are down-regulated in some mutants but up-regulated (highlighted in purple) in others. Genes marked by an asterisk are essential genes based on knockout mutations or RNAi analysis as listed on the Wormbase Web site.
(128 KB XLS)

Table S3 Microarray Data Analysis: Expression Values This table contains the expression values computed from all chips being analyzed. Every chip analysis is named as an alphabetic character followed by a number. X represents *xbp-1(zc12)* mutant, I is *ire-1(vc33),* P is *pek-1(ok275),* A is *atf-6 (RNAi)* and N means wild-type N2 animals. The odd numbers (1, 3, 5) represent samples without tunicamycin treatment, and the even numbers (2, 4, 6) are samples treated with tunicamycin. IA represents *ire-1(v33); atf-6(RNAi)* double mutant animals and IP represents *ire-1(v33); pek-1(ok275).* Because *ire-1(v33); atf-6(RNAi)* and *ire-1(v33); pek-1(ok275)* double mutants were very sick, they were not included in the ANOVA. We included the data from *ire-1(v33); atf-6(RNAi)* and *ire-1(v33); pek-1(ok275)* samples for reference. The expression values and fold changes are represented in log(2) algorithm.
(12.0 MB ZIP)

Table S4 Microarray Data Analysis: Overall Model-Fit Data This table contains 13,706 genes (more exactly, probes) with a *q*-value less than 0.001.
(3.9 MB XLS)

Table S5 Microarray Data Analysis: Interaction Data This table contains 4,050 genes (probes) with an interaction *p*-value less than 0.01. Fold changes were calculated as treated samples versus untreated samples.
(2.2 MB XLS)

Table S6 Microarray Data Analysis: Type Data This table contains significant genes (probes) in the type term (an *F*-value-associated *p*-value less than 0.001). Mean gene expression levels for each strain type (regardless of treatments) were compared and represented as fold changes. Individual *p*-values for each comparison were calculated. The fold change values are red if the corresponding *p*-values are less than 0.005.
(4.6 MB XLS)

Table S7 Genes Up-Regulated in Mutants Significant genes in the type data were filtered by at least 2-fold variance from N2 wild-type samples and significant *p*-values less than 0.005. This table contains genes (probes) that were up-regulated in at least one of the mutants. The significant fold changes are colored red (*p* < 0.005).
(146 KB XLS)

Table S8 *ire-1*-Dependent c-UPR Genes This table summarizes c-UPR genes that are down-regulated in *ire-1(v33)* mutants compared to N2 animals. Fold changes were calculated by comparing the mean expression of mutants including both treated and untreated animals to that of N2 animals. IvN, XvN, PvN, and AvN indicate comparisons of *ire-1, xbp-1, pek-1,* and *atf-6,* respectively, to N2. Genes significantly down-regulated are highlighted in yellow and those up-regulated are in purple (*p* < 0.005). Genes marked by an asterisk are essential genes based on knockout mutations or RNAi analysis as listed on the Wormbase Web site.
(41 KB XLS)

Table S9 *xbp-1-* and/or *atf-6*-Dependent c-UPR Genes This table summarizes c-UPR genes that are down-regulated in either *xbp-1(zc12)* and/or *atf-6 (RNAi)* mutants compared to N2 animals. Fold changes were calculated by comparing the mean expression of mutants including both treated and untreated animals to that of N2 animals. IvN, XvN, PvN, and AvN indicate comparisons of *ire-1, xbp-1, pek-1,* and *atf-6,* respectively, to N2. Genes significantly down-regulated are highlighted in yellow and those up-regulated are in purple (*p* < 0.005). Genes marked by an asterisk are essential genes based on knockout mutations or RNAi analysis as listed on the Wormbase Web site.
(112 KB XLS)

Table S10 Genes Differentially Regulated by *ire-1* and *xbp-1*
Significant genes in the type term were further analyzed by comparing *ire-1* versus *xbp-1* (IvX). Genes (probes) in this spreadsheet had at least a 2-fold change between *ire-1* and *xbp-1* samples and had corresponding *p*-values less than 0.005. The significant fold changes are colored red (*p* < 0.005).
(350 KB XLS)

Table S11 Comparisons to Previous Reported Genes Involved in the ER Stress Response We examined the 26 genes that were reported previously by Urano et al. to be dependent on *xbp-1* signaling for induction upon ER stress [26]. Consistently, 17 genes were also *ire-1/xbp-1-*dependent UPR genes in our array data. However, the expression of four genes either didn't change or showed a down-regulation in an *ire-1/xbp-1-*independent manner upon tunicamycin treatment. LEC-8 showed a less than 2-fold induction in wild-type worms and thus was excluded from our UPR gene list. In addition, the *abu* genes were not up-regulated in either *ire-1* or *xbp-1* mutants. Finally, we looked at *uda-1,* which was reported as an *ire-1-*dependent UPR gene in *C. elegans* by Uccelletti et al. [66]. The expression of *uda-1* was slightly induced in wild-type worms and showed moderate reduction in both *ire-1* and *xbp-1* worms. This result is consistent with the northern blot shown by Uccelletti et al.
(29 KB XLS)

### Accession Numbers

The Wormbase (http://www.wormbase.org/) accession numbers for the *C. elegans* genes and gene products discussed in this paper are *atf-6* (F45E6.2), BAP31 (Y54G2A.18), Ca^2+^-independent phospholipase A2 (F47A4.5), calumenin (M03F4.7), CDK5 activator-binding protein C53 (Y113G7B.16), choline/ethanolaninephosphotransferase (CE05695), CREBh (F57B10.1), Derlin-1-interacting AAA ATPase p97 (C41C4.8), EDEM (C47E12.3), eIF2C4 (C04F12.1), Herp (F25D7.2), HRD1 (F55A11.3), nucleobindin (F44A6.1), Rer1 (F46C5.8), and S2P (Y56A3A.2). The UniGene (http://www.ncbi.nlm.nih.gov/entrez/query.fcgi?db=unigene) accession number for mammalian *CREBh* is Hs.247744.

## Abbreviations

ANOVA: analysis of variance
bZIP: basic leucine zipper
c-UPR: constitutive unfolded protein response
eIF2α: the alpha subunit of eukaryotic translation initiation factor 2
ER: endoplasmic reticulum
ERAD: endoplasmic reticulum–associated degradation
i-UPR: inducible unfolded protein response
GFP: green fluorescent protein
MEF: mouse embryonic fibroblast
PC: phosphatidylcholine
RNAi: RNA interference
S1P: site-1 protease
S2P: site-2 protease
UPR: unfolded protein response
