# Supplementary material for: Genetic Interactions Due to Constitutive and Inducible Gene Regulation Mediated by the Unfolded Protein Response in C. elegans
Source: PLoS Genet. 2005 Sep 23;1(3):e37. doi: 10.1371/journal.pgen.0010037 (PMC1231716; doi:10.1371/journal.pgen.0010037)
Supplement: Supplementary file 1 [file pgen.0010037.sg001.pdf]

Supplemental Figure 1

A

tunicamycin 5 ug/ml	*atf-6(ok551)*			N2		
	a	b	c	a	b	c
eggs laid	263	393	457	288	479	602
addults	153	150	68	76	134	138
percentage	58%	38%	15%	26%	28%	23%

B

tunicamycin 7.5 ug/ml	*atf-6(ok551)*		N2	
	a	b	a	b
eggs laid	178	386	174	338
addults	23	30	4	5
percentage	13%	8%	2%	1%
