# Supplementary material for: Genetic Interactions Due to Constitutive and Inducible Gene Regulation Mediated by the Unfolded Protein Response in C. elegans
Source: PLoS Genet. 2005 Sep 23;1(3):e37. doi: 10.1371/journal.pgen.0010037 (PMC1231716; doi:10.1371/journal.pgen.0010037)
Supplement: Supplementary file 2 [file pgen.0010037.sg002.pdf]

Supplementary Figure 2

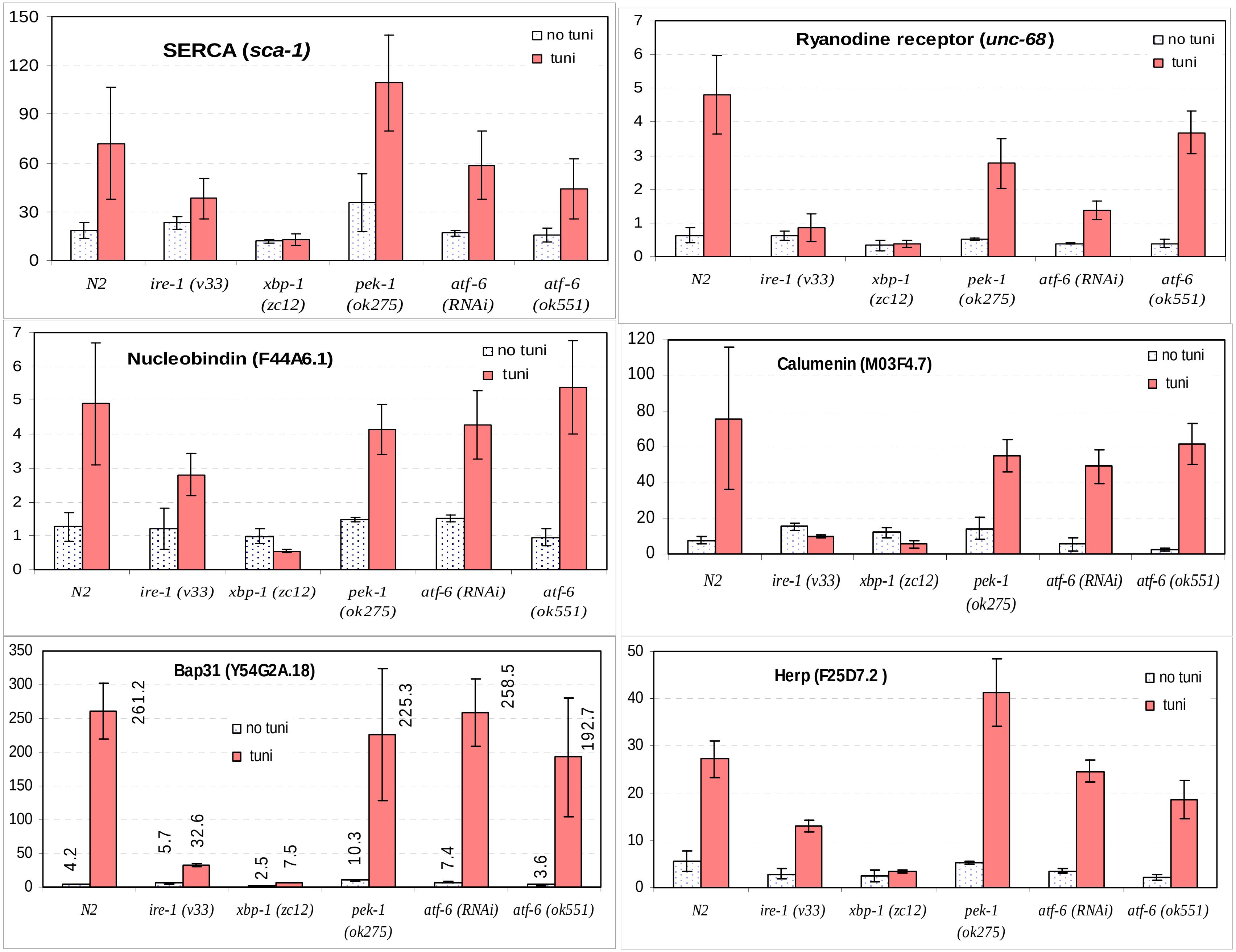

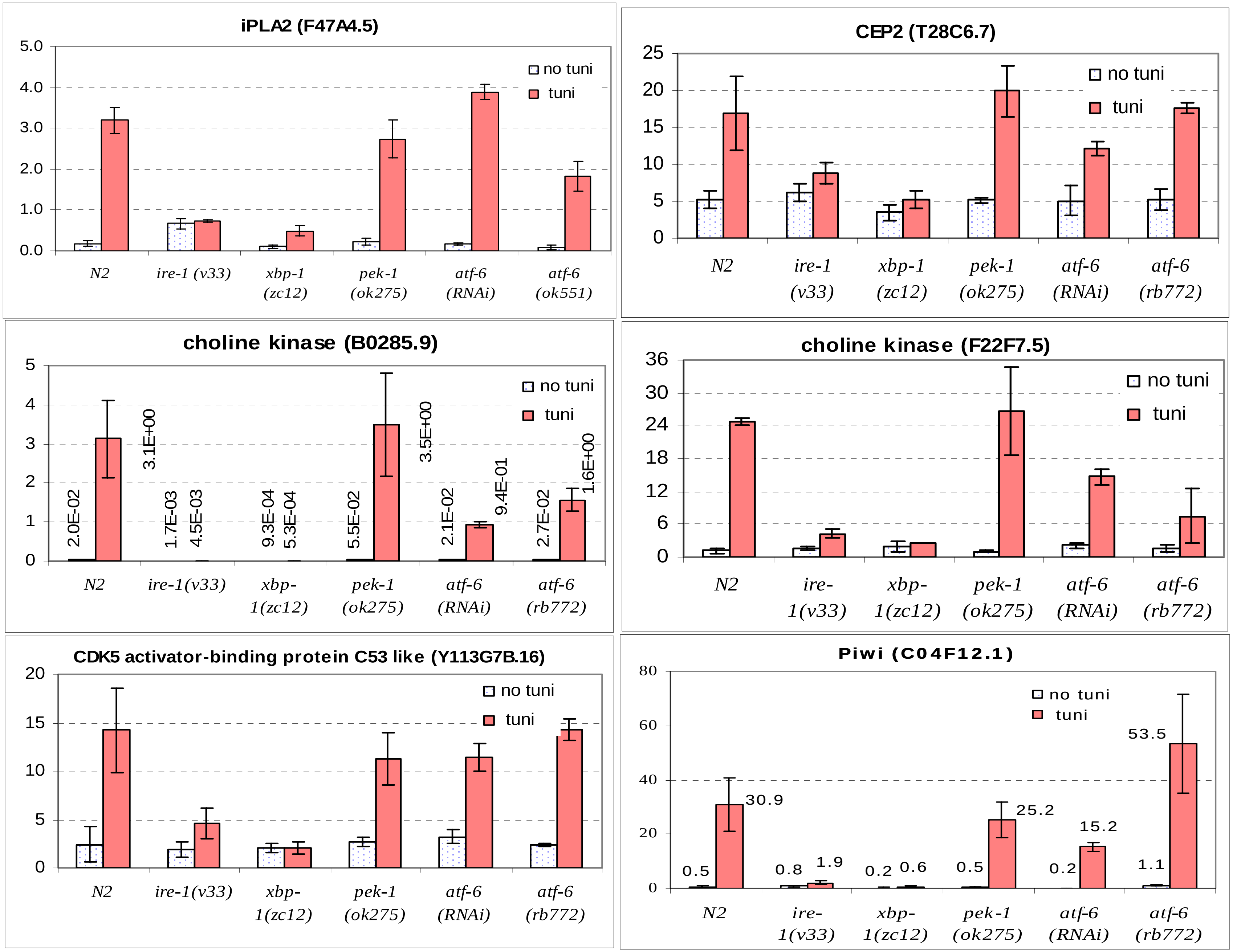

iPLA2 (F47A4.5)
5.0
4.0
3.0
2.0
1.0
0.0
no tuni
tuni
N2
ire-1 (v33)
xbp-1 (zc12)
pek-1 (ok275)
atf-6 (RNAi)
atf-6 (ok551)
CEP2 (T28C6.7)
25
20
15
10
5
0
no tuni
tuni
N2
ire-1 (v33)
xbp-1 (zc12)
pek-1 (ok275)
atf-6 (RNAi)
atf-6 (rb772)
choline kinase (B0285.9)
5
4
3
2
1
0
no tuni
tuni
2.0E-02
3.1E+00
1.7E-03
4.5E-03
9.3E-04
5.3E-04
5.5E-02
3.5E+00
2.1E-02
9.4E-01
2.7E-02
1.6E+00
N2
ire-1(v33)
xbp-1(zc12)
pek-1 (ok275)
atf-6 (RNAi)
atf-6 (rb772)
choline kinase (F22F7.5)
36
30
24
18
12
6
0
no tuni
tuni
N2
ire-1(v33)
xbp-1(zc12)
pek-1 (ok275)
atf-6 (RNAi)
atf-6 (rb772)
CDK5 activator-binding protein C53 like (Y113G7B.16)
20
15
10
5
0
no tuni
tuni
N2
ire-1(v33)
xbp-1(zc12)
pek-1 (ok275)
atf-6 (RNAi)
atf-6 (rb772)
Piwi (C04F12.1)
80
60
40
20
0
no tuni
tuni
0.5
30.9
0.8
1.9
0.2
0.6
0.5
25.2
0.2
15.2
1.1
53.5
N2
ire-1(v33)
xbp-1(zc12)
pek-1 (ok275)
atf-6 (RNAi)
atf-6 (rb772)

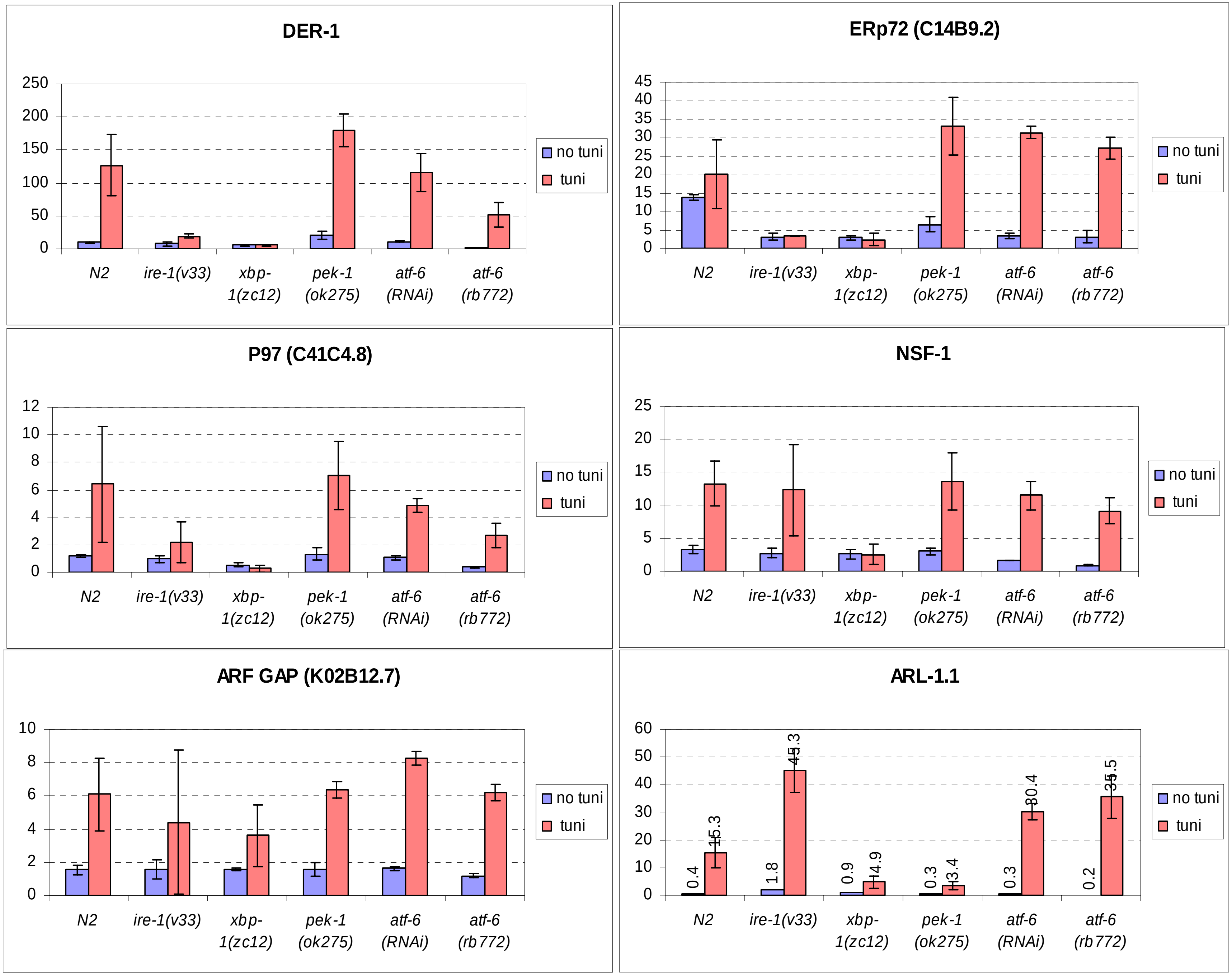
DER-1
250
200
150
100
50
0
no tuni
tuni
N2
ire-1(v33)
xbp-1(zc12)
pek-1 (ok275)
atf-6 (RNAi)
atf-6 (rb772)
ERp72 (C14B9.2)
45
40
35
30
25
20
15
10
5
0
no tuni
tuni
N2
ire-1(v33)
xbp-1(zc12)
pek-1 (ok275)
atf-6 (RNAi)
atf-6 (rb772)
P97 (C41C4.8)
12
10
8
6
4
2
0
no tuni
tuni
N2
ire-1(v33)
xbp-1(zc12)
pek-1 (ok275)
atf-6 (RNAi)
atf-6 (rb772)
NSF-1
25
20
15
10
5
0
no tuni
tuni
N2
ire-1(v33)
xbp-1(zc12)
pek-1 (ok275)
atf-6 (RNAi)
atf-6 (rb772)
ARF GAP (K02B12.7)
10
8
6
4
2
0
no tuni
tuni
N2
ire-1(v33)
xbp-1(zc12)
pek-1 (ok275)
atf-6 (RNAi)
atf-6 (rb772)
ARL-1.1
60
50
40
30
20
10
0
0.4
15.3
1.8
45.3
0.9
4.9
0.3
3.4
0.3
30.4
0.2
35.5
no tuni
tuni
N2
ire-1(v33)
xbp-1(zc12)
pek-1 (ok275)
atf-6 (RNAi)
atf-6 (rb772)

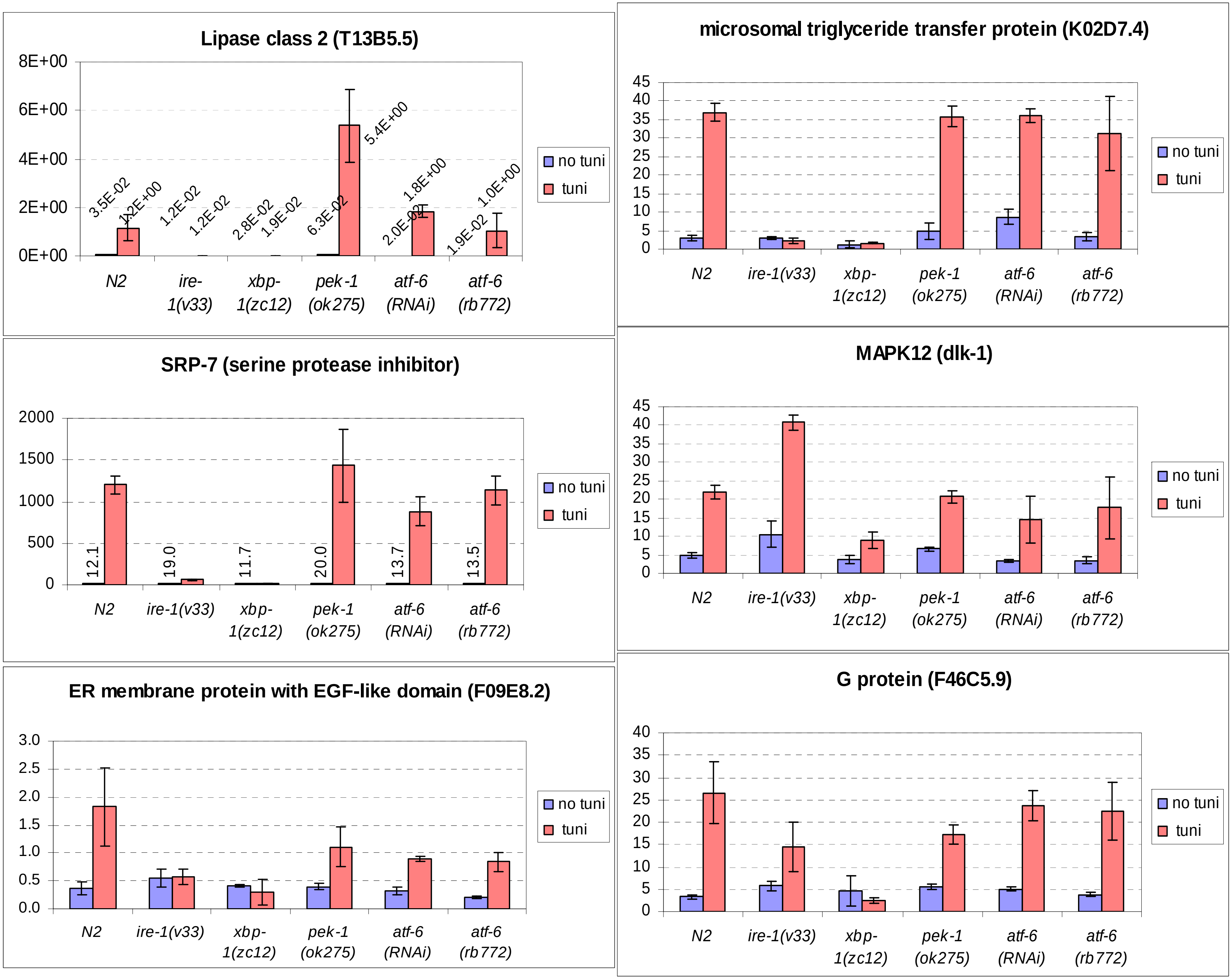

Lipase class 2 (T13B5.5)
8E+00
6E+00
4E+00
2E+00
0E+00
3.5E-02
1.2E+00
1.2E-02
1.2E-02
2.8E-02
1.9E-02
6.3E-02
5.4E+00
2.0E-03
1.8E+00
1.9E-02
1.0E+00
N2
ire-1(v33)
xbp-1(zc12)
pek-1 (ok275)
atf-6 (RNAi)
atf-6 (rb772)
no tuni
tuni
microsomal triglyceride transfer protein (K02D7.4)
45
40
35
30
25
20
15
10
5
0
N2
ire-1(v33)
xbp-1(zc12)
pek-1 (ok275)
atf-6 (RNAi)
atf-6 (rb772)
no tuni
tuni
SRP-7 (serine protease inhibitor)
2000
1500
1000
500
0
12.1
19.0
11.7
20.0
13.7
13.5
N2
ire-1(v33)
xbp-1(zc12)
pek-1 (ok275)
atf-6 (RNAi)
atf-6 (rb772)
no tuni
tuni
MAPK12 (dlk-1)
45
40
35
30
25
20
15
10
5
0
N2
ire-1(v33)
xbp-1(zc12)
pek-1 (ok275)
atf-6 (RNAi)
atf-6 (rb772)
no tuni
tuni
ER membrane protein with EGF-like domain (F09E8.2)
3.0
2.5
2.0
1.5
1.0
0.5
0.0
N2
ire-1(v33)
xbp-1(zc12)
pek-1 (ok275)
atf-6 (RNAi)
atf-6 (rb772)
no tuni
tuni
G protein (F46C5.9)
40
35
30
25
20
15
10
5
0
N2
ire-1(v33)
xbp-1(zc12)
pek-1 (ok275)
atf-6 (RNAi)
atf-6 (rb772)
no tuni
tuni

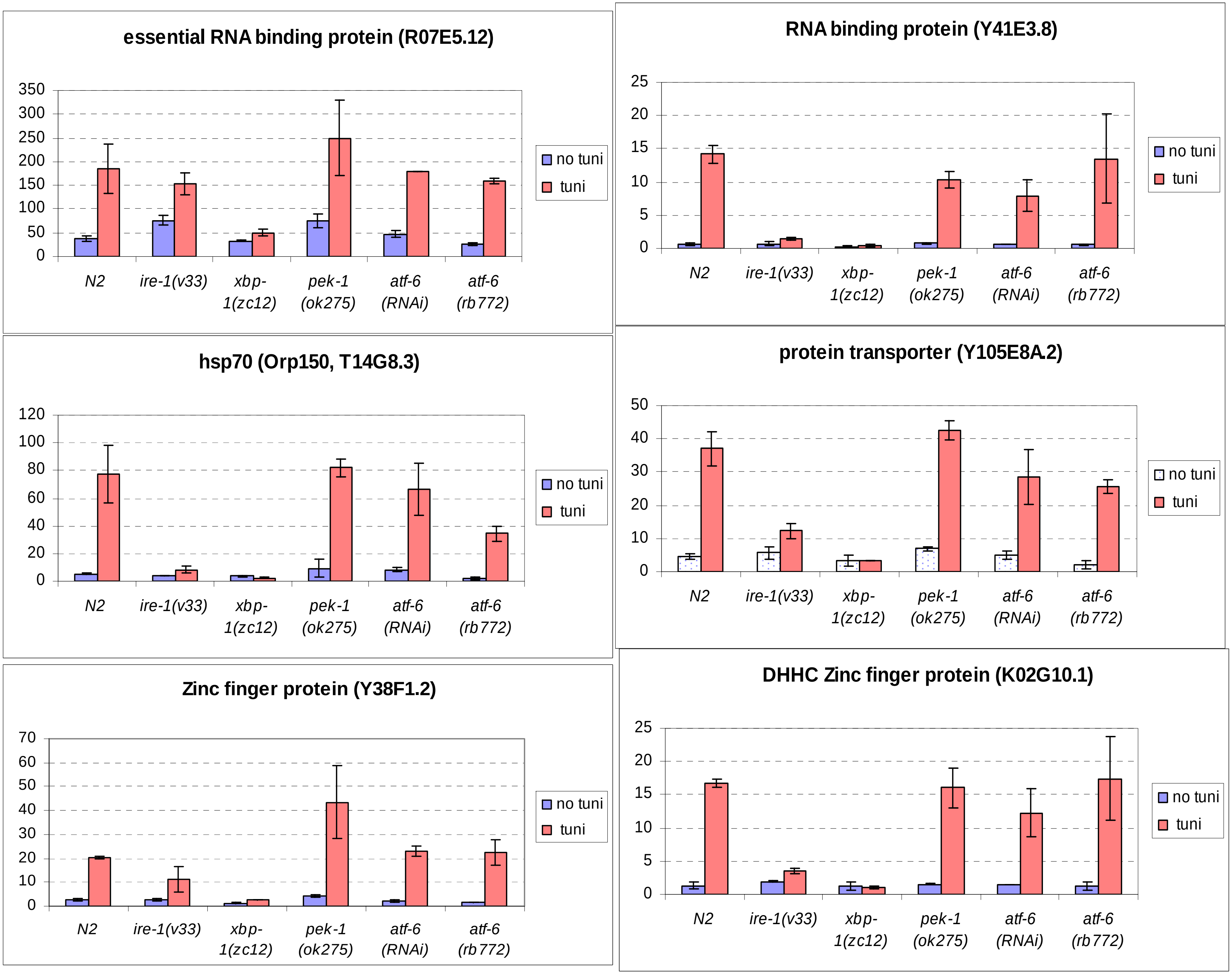
essential RNA binding protein (R07E5.12)
RNA binding protein (Y41E3.8)
hsp70 (Orp150, T14G8.3)
protein transporter (Y105E8A.2)
Zinc finger protein (Y38F1.2)
DHHC Zinc finger protein (K02G10.1)
no tuni
tuni
N2
ire-1(v33)
xbp-1(zc12)
pek-1 (ok275)
atf-6 (RNAi)
atf-6 (rb772)
