# Supplementary material for: Genetic Interactions Due to Constitutive and Inducible Gene Regulation Mediated by the Unfolded Protein Response in C. elegans
Source: PLoS Genet. 2005 Sep 23;1(3):e37. doi: 10.1371/journal.pgen.0010037 (PMC1231716; doi:10.1371/journal.pgen.0010037)

Supplementary Figure 3

A. *pek-1* promoted GFP

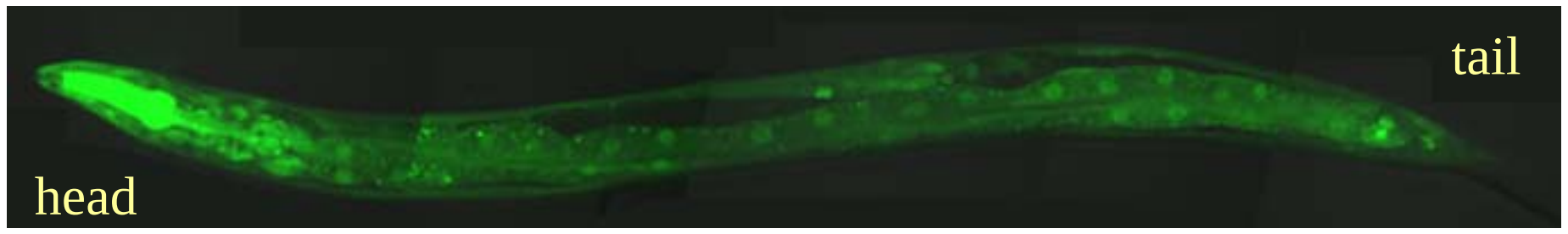

B. *atf-6* promoted GFP

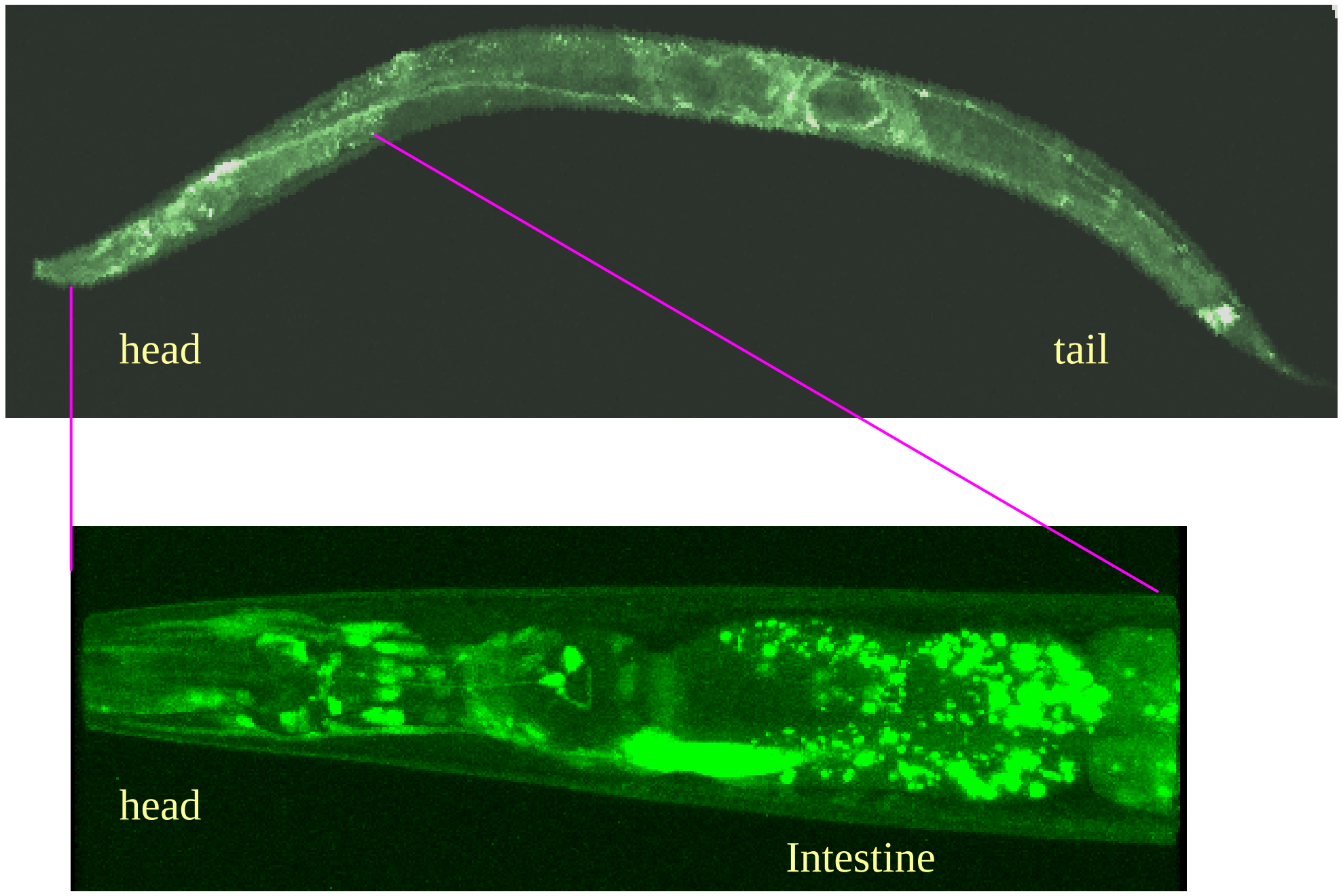

Supplement: Supplementary file 3 [file pgen.0010037.sg003.pdf]
